# Investigation of Gender Stereotypes in Nurse Clinicians' Metaphors and Concepts of Patients

**DOI:** 10.1155/2024/8861439

**Published:** 2024-11-15

**Authors:** Selver Bezgin, Özge Odabaşı Koç

**Affiliations:** ^1^Faculty of Health Sciences, Department of Mental Health and Diseases Nursing, Van Yüzüncü Yıl University, Van, Türkiye; ^2^Van Fine Arts High School, Van, Türkiye

**Keywords:** gender, metaphor, nurse clinician, patient

## Abstract

**Aim:** The present study aims to analyze the presence of gender stereotypes in the metaphors nurse clinicians use to describe their patients.

**Methods:** This study was conducted with 149 nurse clinicians working at a university hospital. Data were collected using a metaphor survey.

**Results:** The most common metaphors for female patients were “flower” (f: 23) and “mother/my mother” (f: 8), whereas the most common metaphors for male patients were “wood” (f: 10) and “cactus” (f: 7). These metaphors were more frequently produced by female nurses. Female patients were often described as “delicate, in need of attention and help, and fragile,” while male patients were described as “strong, authoritative, head of the family, and tough.” Female nurses likened a more delicate male patient to a “delicate woman,” and male nurses likened the strength of a male patient to a “man.” Furthermore, eight nurses, five male nurses and three female nurses, frequently used the metaphor of “patient” (f: 6) while stating that they did not view patients as female or male. Male nurses (f: 4) produced the metaphor of “patient” (f: 6) the most.

**Conclusion:** It is important not to overlook the factors underlying the metaphors produced by nurse clinicians. In this regard, extensive studies are needed to take into account additional factors such as cultural background, experience, or specific patient interactions that may have a major impact on how nurses perceive gender.

**Implications for Nursing Management:** This study evaluates nurse clinicians' perceptions of patients in the context of gender stereotypes and highlights some important points in terms of nursing and patient care.

## 1. Introduction

Gender refers to the roles assigned to women and men by society. These roles vary over time, and they differ from one society to another. Considering that these roles are assigned by society, unequal roles, such as being more active at home and passive in one's work life, are assigned to women; traditional roles, such as being active outside the home and being the head of the family, are assigned to men [1–3]. Society assigns these roles to men and women even before they are born, and these roles become stereotypes. Gender roles are also produced and transmitted as a result of these stereotypes [2, 4]. These stereotypes in society generally depict women as warm, loving, sensitive, delicate, and compassionate. On the other hand, men are seen as independent, dominant, and strong. These gender stereotypes usually cause one gender to remain behind, leading to inequality [5, 6]. In some societies, these inequalities can exist not only within the healthcare system, but in the work, family, and social spheres as well. Although healthcare services are generally delivered in an egalitarian manner, gender role stereotypes have been seen to lead to inequalities in this delivery [2, 4, 7]. However, healthcare professionals, and particularly nurses, who are in constant interaction with patients, should avoid gender stereotypes and provide individuals with an egalitarian perspective on gender roles [2, 4, 8].

Nurses' gender stereotypes can impact their approach to the individuals they care for and, therefore, the quality of nursing care [9–13]. A recent study examined the effects of team reflexivity and gender role attitudes on the intention of female nurses to continue working; the findings suggested that team-based self-esteem (i.e., as opposed to team reflexivity, which nurses perceive individually) has more of a positive effect on nurses' intention to continue to work. This effect, however, was weakened by traditional gender role attitudes [14]. Another study conducted on nurses determined that traditional gender role attitudes have negative effects on a nurse's intention to continue working and their level of cooperation with physicians. This same study also found that nurses who adopted traditional gender role attitudes were more susceptible to burnout and less likely to enhance the quality of the nursing care they provided [10]. The literature reports that traditional gender role attitudes may reduce a nurse's sense of responsibility toward patients and doctors. This may ultimately reduce the sense of professional autonomy in nurses due to an acceptance of a subservient relationship with the medical profession. The literature has also noted that egalitarian gender roles tend to yield better responses, such as flexible working, healthier behavior, and less stress. These outcomes are significant for nurses, as they carry the greatest burden in healthcare services, and they also assume more responsibility when it comes to providing healthcare, increasing the quality of care, and improving the institution [10].

Gender stereotypes not only adversely affect the quality of nursing care but also negatively impact nurses' private lives. The literature states that traditional gender role attitudes indicate some inconsistency between self-concept and social expectations. Such inconsistency can create a negative emotional state in individuals and therefore trigger depression and anxiety. Furthermore, it is stated that female nurses with traditional gender role attitudes are more likely to feel conflicted from the pressures of work and home than female nurses with modern gender role attitudes and support within the team. However, the sustainability of this support may weaken due to the struggle between personal roles and social expectations [14].

## 2. Aim

The present study aims to analyze gender stereotypes via the metaphors nurse clinicians use to describe their patients. The purpose of using metaphors in data collection is to obtain in-depth information about whether nurses have gender stereotypes while caring for patients. The literature states that a situation that is hidden from or not entirely known to individuals can be revealed in depth through metaphors [15].

### 2.1. Research Questions

In line with the study's aim, answers to the following questions were sought:

1. What metaphors do nurse clinicians use to describe their concept of a patient?
2. Do the metaphors that nurse clinicians use to describe their concept of a patient differ in terms of gender stereotypes?
3. Do the metaphors determined by nurse clinicians for the concept of patient differ by gender in terms of gender stereotypes?

## 3. Methods

### 3.1. Research Design

This study used a basic qualitative research design. This design was preferable because mental images (metaphors) are not an observable situation; these images cannot be examined under a microscope and, therefore, are better understood through nurse clinicians' descriptions [16].

To determine the study group, the purposive sampling method of homogeneous sampling was used. Nurses from the same province and with similar working conditions were selected. The homogeneous sampling method is employed to define a distinct subgroup by creating a small and homogeneous sample [15].

### 3.2. Research Team

The research team consisted of two researchers: a nursing faculty member (PhD, assistant professor) in the Mental Health and Disease Department and an expert philosophy teacher (MSc) at a fine arts high school. One researcher was 46 years-old and the other was 36 years-old. Both researchers were female and trained in the area of qualitative research. They were also acquainted with all of the participants.

### 3.3. Determination and Identification of Participants

The research was conducted in a university hospital in XXX, where 611 nurses work. Considering the inclusion and exclusion criteria, a survey and a metaphor form were given to 167 nurses. Forms from 18 nurses, 10 female and 8 male nurses, who had incompatibility between metaphor and explanation (n: 15) or who left the metaphor field or field for an explanation of the metaphor blank (n :3) were excluded from the analysis. Therefore, the forms of 149 nurses were evaluated.

#### 3.3.1. Inclusion Criteria

• When selecting the sample, care was taken to ensure that nurses working in different wards participated in the study.

#### 3.3.2. Exclusion Criteria

The following nurses were not included in the study:

• Nurses working in departments with limited communication with patients (such as the operating room).
• Nurses working in departments with pediatric patients (e.g., pediatric ward and pediatric emergency service).
• Nurses working in departments only with male or female patients (e.g., urology ward and obstetrics and gynecology ward).

The mean age of the nurses included in the study was 31.14 ± 6.826 years. These nurses had worked for an average of 9.54 ± 6.466 years. The number of female patients was 82 (55%), while the number of male patients was 67 (45%).  Table 1 lists other descriptive characteristics of the participants.

### 3.4. Data Collection Tools

The research data were collected using a personal information form prepared by the researchers. The form contained demographic information such as age, gender, educational background, ward of employment, number of years employed, and a metaphor form consisting of semistructured questions.

#### 3.4.1. Metaphor Form

In the semistructured metaphor form, each nurse clinician was asked to complete the sentences, “A female patient is like… because…” and “A male patient is like… because….” The literature states that metaphors alone cannot adequately reveal the descriptive and visual power of a situation, so the question “why” must also be asked [15]. Hence, we asked nurse clinicians to complete the sentence starting with “because…” to explain the meaning behind the metaphors used for the concepts of “female patient” and “male patient.” In doing so, we aimed to perform an in-depth examination of these metaphors in the context of gender stereotypes.

### 3.5. Implementation of Data Collection Tools

Prior to data collection, the researchers obtained ethical permission as well as permission from the institution where the research data would be collected. The research data were collected between December 16, 2023 and January 30, 2024. The researchers reached the nurses who volunteered for the study during their work hours, and a purposive sampling method was used. The nurses were informed about the study, and they gave their consent. They were then asked to fill out the survey and metaphor form through self-reporting.

### 3.6. Data Analysis

Frequencies and percentages among the participants' descriptive statistics were analyzed using IBM SPSS Statistics for Windows, Version 23.0.

Answer sheets collected with the metaphor form were used as the main data source in the study. The data obtained were evaluated using the qualitative method of content analysis. The data analysis began with checking whether the nurse clinicians' answer sheets were completed appropriately. Those who appropriately filled in the sections on the subject of the metaphor and the source of the metaphor were evaluated. Next, the nurses' answer sheets were numbered from 1 to 149. Content analysis grouped similar data together within the framework of certain concepts and categories. The findings section presents the nurses' opinions, along with an indicator made up of each nurse's answer sheet number and gender. For example, a female nurse with the sheet number “49” was given the indicator “FN, 49.” Similarly, a male nurse with sheet number 93 was given the indicator “MN, 93.” The content analysis grouped metaphors and metaphor explanations with certain common features under the same category. For example, the metaphors “flower,” “ivy,” and “daisy” were grouped under the “plant” category, whereas the metaphors “bear,” “ant,” and “hyena” were grouped under the “animal” category. The nurses' metaphors also included the following explanations: “…easily hurt and sensitive,” “…expect constant care and attention,” “…fragile,” and “…need help and love.” These explanations were collected under the “delicate, in need of attention and help, and fragile” category. Other categories were also created in this way, and frequency analysis was used to determine the frequencies of these metaphors and categories. “Frequency analysis reveals the frequency of units or elements in numerical form. This provides an understanding of the intensity and importance of a particular element” [17]. For example, a total of 23 nurses produced the metaphor “flower” for female patients, shown as “flower” (f: 23) in this study.

As a result, the process of analyzing and interpreting the metaphors produced by nurse clinicians consisted of the following stages:

• Examination of answer sheets
• Elimination of unsuitable answer sheets
• Recompilation of answer sheets
• Sorting and numbering of answer sheets
• Examination of metaphors
• Development of categories
• Distribution of metaphors into categories
• Ensuring validity and reliability
• Calculation of the frequencies of metaphors
• Interpretation of data

### 3.7. Ensuring Validity and Reliability

The following processes were carried out to ensure the validity and reliability of the study.

#### 3.7.1. For Validity

• Purposive sampling was used.
• Inclusion/exclusion criteria were determined.
• The participants were introduced in detail.
• For each category obtained in the study, examples that were assumed to best represent the nurses were selected from their statements and included in the findings section.

#### 3.7.2. For Reliability

A detailed literature review was performed in order to ensure consistency between the relevant studies.

• The opinions of two qualitative research method experts were obtained, and it was concluded that the categories determined were highly consistent.

### 3.8. Ethical Considerations

The present study was conducted in accordance with the principles of the Declaration of Helsinki. Prior to the study, written permission was obtained from the Noninterventional Clinical Research Ethics Committee of Van Yuzuncu Yil University (ethics approval number: 2023/13-01; date: 12-08-2023) and the institution where the study was conducted. Prior to commencing the study, the research was explained to the participants, who then gave their consent and agreed to participate in the study.

## 4. Results

This section includes findings obtained through metaphor analysis, as well as direct quotations regarding these metaphors.

### 4.1. Findings on Metaphors Used for Female Patients

This subsection includes the metaphors, explanations, and direct quotations from the participants with regard to female patients.  Table 2 contains the categories obtained from the metaphors and metaphor explanations.

As seen in Table 2, the metaphors participants used for female patients were classified into nine categories. The highest number of metaphors for female patients was produced in the qualitative (f: 19) category. The “flower” (f: 23) metaphor, frequently repeated for female patients, was mostly produced by female nurses (f: 16). With regard to female patients, female nurses produced 54 metaphors, while male nurses produced 50 metaphors.

As seen in Table 2, the participants' metaphor explanations were classified into 15 categories. The findings for each of these categories are presented as follows.

#### 4.1.1. Category 1. Family Member

Eight nurse clinicians produced 4 metaphors in this category. The most used metaphor in this category, “mother/my mother” (f: 5), was produced by 3 female nurses and 2 male nurses. “Aunt” (f: 2) and “elder sister” were other metaphors produced in this category.

A female patient is like a mother because I work in the medical oncology ward. When I see women with cancer who present to the ward and their poor prognosis, I think of my mother (FN, 80).

A female patient is like a mother because expressions on her face and wear and tear on her body remind me of what my mother went through (FN, 81).

A female patient is like a mother because every woman reminds me of my mother (MN, 145).

#### 4.1.2. Category 2. Delicate, in Need of Attention and Help, and Fragile

A total of 33 metaphors were produced in this category; 33 female nurses and 29 male nurses produced metaphors describing female patients as “delicate, in need of attention and help, and fragile.” This category had the highest number of metaphors and responses from the nurse clinicians. The most frequently repeated metaphor in this category, “flower” (f: 20), was used by female nurses (f: 14).

A female patient is like a glass because she is easily hurt and sensitive (FN, 30).

A female patient is like a girl because she is innocent, needy, and unprotected (FN, 50).

A female patient is like a flower because she is delicate and fragile. The patient profile in our hospital is particularly hungry for love and attention, and they love to show coyness to their husbands (FN, 68).

A female patient is like a car because she breaks down when neglected in every aspect (MN, 101).

A female patient is like a new bride because she is very coy (MN, 103).

A female patient is like a wounded bird because she needs help and love (MN, 106).

A female patient is like a child whose toy was taken away because she is very coy and fragile (MN, 130).

#### 4.1.3. Category 3. Thinking About Her Responsibilities at Home but Neglecting Herself

Ten nurse clinicians produced 9 metaphors in this category. The most frequently used metaphor in this category, “suffering” (f: 2), was produced by female nurses.

A female patient is like a poor person because she has numerous responsibilities and thinks about other people and situations even when sick (FN, 22).

A female patient is like a mother because the burden on women is greater (FN, 78).

A female patient is like a wounded lion because she doesn't even realize that she is worn out after years of looking after children, tidying up the house, and cooking (MN, 126).

A female patient is like a candle because she illuminates the surroundings (MN, 132).

#### 4.1.4. Category 4. Dressed up in Layers

Two nurse clinicians, both of whom were female, produced two metaphors in this category: “cabbage” and “lettuce.”

A female patient is like a cabbage because she dresses in layers (FN, 58).

A female patient is like lettuce because she dresses in layers due to the fear of getting sick and being very cold (FN, 21).

#### 4.1.5. Category 5. Keeping Alive and Directing

Five nurse clinicians produced 5 metaphors in this category. Female nurses produced the metaphors of “earth,” “tree,” and “streambed,” while male nurses produced the metaphors of “bird” and “ant.”

A female patient is like Earth because she keeps everyone alive and gathers them together (FN, 5).

A female patient is like a streambed because she directs everything (FN, 24).

A female patient is like an ant because a woman is life. She is a mother, elder sister, sister, wife when necessary. There is peace in her presence and loneliness in her absence (MN, 90).

A female patient is like a bird because her nest is destroyed when she dies (MN, 111).

#### 4.1.6. Category 6. Precious, Fun, and Beautiful

Six nurse clinicians produced 6 metaphors in this category. Only female nurses produced metaphors in this category. The metaphors of “sacred being,” “tulumba dessert,” “game,” “sky,” “swan,” and “beautiful mother” were produced.

A female patient is like a sacred being because she is a precious being that must be respected in terms of her own existence, the reason for her creation, her function in the world, and sexuality (FN, 28).

A female patient is like a tulumba dessert because she is very cute and sweet (FN, 41).

A female patient is like the sky because she is very bright, beautiful, and broad (FN, 53).

#### 4.1.7. Category 7. Strong and Resilient

Six nurse clinicians produced 5 metaphors in this category. Female nurses produced the most metaphors in this category (f: 5). Female nurses produced the metaphors of “mountain” (f: 2), “lion,” “ballpoint pen,” and “mother,” whereas male nurses produced the metaphor of “withered rose.”

A female patient is like a mountain because she withstands all kinds of pain (FN, 9).

A female patient is like a lion because she is very strong and resilient (FN, 29).

A female patient is like a ballpoint pen because her energy never runs out (FN, 35).

A female patient is like a withered rose because only disease can destroy her (MN, 109).

#### 4.1.8. Category 8. Easy-going and Leaving Things to Experts

Three nurse clinicians produced 3 metaphors in this category. Female nurses produced the metaphors of “cotton” and “ostrich,” while male nurses produced the metaphor of “fair.”

A female patient is like cotton because she does whatever we say (FN, 36).

A female patient is like a fair person because she treats everyone equally (MN, 124).

#### 4.1.9. Category 9. Clean and Active in Her Care

Four nurse clinicians produced the metaphors “flower” and “white” in this category. “Flower” (f: 3), the most used metaphor in this category, was produced by 2 female nurses and 1 male nurse. One female nurse produced the metaphor of “white.”

A female patient is like something white because female patients are more active in their care and more friendly (FN, 60).

A female patient is like a flower because female patients can actively participate and pay attention to their care. They keep their rooms clean (FN, 75).

A female patient is like a flower because she is very clean and quiet (MN, 134).

#### 4.1.10. Category 10. Harsh, Dissatisfied, and Angry

Eighteen nurse clinicians produced 17 metaphors in this category. Female nurses produced the metaphors of “cactus,” “winter,” “mother-in-law,” “dynamite ready to explode,” “dissatisfied,” “naughty child,” “cat,” “psychiatric patient,” “tape recorder,” “pressure cooker,” “fire,” and “thorn.” Male nurses produced the metaphors of “cactus,” “water,” “torture,” “poison,” “coy child,” and “hypochondriac.”

A female patient is like a cat because she is ill-tempered and stubborn (FN, 39).

A female patient is like a tape recorder because she repeats her wishes and complains all the time (FN, 43).

A female patient is like a mother-in-law because she is more sick and dissatisfied than a man (FN, 16).

A female patient is like a hypochondriac because some part of her body aches all the time (MN, 119).

A female patient is like a cactus because she is very sharp and exposes her thorns when she gets angry (MN, 120).

A female patient is like poison because she is disgusting (MN, 143).

#### 4.1.11. Category 11. The Patient has No Gender

Eight nurse clinicians produced 3 metaphors in this category. “Patient” (f: 6), the most used metaphor in this category, was mostly produced by male nurses (f: 4). In addition, 1 female nurse produced the metaphor of “baby,” and 1 male nurse produced the metaphor of “my own child.”

A female patient is like a baby because we care for and support her the same way we care for a child or baby. I have the same opinion for female and male patients (FN, 32)

A female patient is like a patient because I look at her like a patient, not like a woman (FN, 65).

A female patient is like a patient because the patient has no gender (MN, 100).

A female patient is like my own child because you love her, get angry with her, but you have to fulfill your responsibilities toward her (MN, 139).

#### 4.1.12. Category 12. Private

Four nurse clinicians produced 4 metaphors in this category. Only male nurses produced metaphors in this category. The metaphors produced were “sensitive,” “red flower,” “mother,” and “inverted tulip.”

A female patient is like a mother because her privacy should be taken into consideration (MN, 136).

A female patient is like an inverted tulip because women's privacy is attached to great importance in my region. This requires attention (MN, 147).

#### 4.1.13. Category 13. Weak and Unable to Recover When Sick

Two nurse clinicians produced 3 metaphors in this category. A female nurse produced the metaphor of “suicide,” whereas male nurses produced the metaphors of “fallen leaf” and “bird with a broken wing.”

A female patient is like suicide because it is difficult for women to live after the difficulties they have experienced (FN, 6).

A female patient is like a fallen leaf because she is more stressed and weak (MN, 98).

#### 4.1.14. Category 14. Difficult to Understand and Unable to Express Herself

Six nurse clinicians produced 6 metaphors in this category. Female nurses produced the metaphors of “glass ceiling” and “matryoshka,” while male nurses produced the metaphors of “bell jar,” “time,” “trouble,” and “ashura.”

A female patient is like a glass ceiling because, unfortunately, she has difficulty expressing herself, especially in our country, amid crushed and repressed emotions (FN, 18).

A female patient is like a bell jar because she is shy and cannot express herself (MN, 108).

A female patient is like a trouble because it is more difficult to understand women than to understand life (MN, 129).

#### 4.1.15. Category 15. Other

Three nurse clinicians, all of whom were male, produced 3 metaphors in this category: “mirror,” “irregular,” and “burden.”

A female patient is like a mirror because she treats you the way you treat her (MN, 87).

A female patient is like a burden because she is very heavy (MN, 89).

A female patient is like an irregular person because she doesn't pay attention to hygiene (MN, 92).

### 4.2. Findings Regarding the Metaphors Produced for Male Patients

This subsection includes the categories of metaphors, explanations, and direct quotations from participants with regard to male patients.  Table 3 contains the categories obtained from the metaphors and metaphor explanations. As seen in Table 3, the metaphors produced by the participants were classified into 10 categories. The highest number of metaphors for male patients was produced in the qualitative (f: 29) category. “Wood” (f: 10), the most frequently repeated metaphor for male patients, was mostly produced by female nurses (f: 8). For male patients, female nurses produced 58 metaphors, while male nurses produced 56 metaphors. Only male nurses produced metaphors in the “food/beverage” category.

As seen in Table 3, the explanations of the participants' metaphors for male patients were classified into 15 categories. The findings from these categories are as follows.

#### 4.2.1. Category 1. Family Member

Seven nurse clinicians produced 3 metaphors in this category. The most used metaphor in this category, “father/my father” (f: 4), was produced by 3 female nurses and 1 male nurse. Female nurses also produced the metaphors of “grandfather/my grandfather” (f: 2) and “uncle.”

A male patient is like my grandfather because I empathize with him (FN, 37).

A male patient is like an uncle because he reveals my sense of compassion by approaching with empathy (FN, 40).

A male patient is like my father because my father suffered from diseases a lot. He always comes to my mind (MN, 147).

#### 4.2.2. Category 2. Easy-Going and Leaving Things to Experts

Seven nurse clinicians produced 7 metaphors in this category. There were no repeated metaphors. The metaphors of “positive motivation” and “chameleon” were produced by female nurses, whereas the metaphors of “gentleman,” “sugar,” “dead,” “fellow man,” and “honey” were the metaphors produced by male nurses.

A male patient is like positive motivation because he leaves things to experts (FN, 17).

A male patient is like a dead person because he doesn't object to anything during his stay (MN, 110).

A male patient is like a fellow man because he leaves things to experts and does not interfere in everything (MN, 129).

#### 4.2.3. Category 3. Strong, Authoritative, Head of the Family, and Resilient

Twenty nurse clinicians produced 16 metaphors in this category. Both female and male nurses produced the metaphors of “iron” (f: 2), “cactus” (f: 2), and “mountain” (f: 2). In addition, female nurses produced the metaphors of “authority,” “bully,” “elder brother,” “strong father,” “stone,” and “lion” (f: 2). Male nurses produced the metaphors of “resilient,” “ant,” “man,” “father,” “Google,” “ignorant wise man,” and “column of the building.”

A male patient is like an authority because he's trying to be ruder and more dominant (FN, 18).

A male patient is like a bully because he has high expectations and tries to get what he says (FN, 27).

A male patient is like an elder brother because he is strong and brave (FN, 79).

A male patient is like a cactus because he can stay without water for years (MN, 107).

A male patient is like a man because he is strong (MN, 128).

A male patient is like a father because I think he is a little more resilient and heavier (MN, 145).

#### 4.2.4. Category 4. Rude, Dissatisfied, and Angry

Thirty-two nurse clinicians produced 25 metaphors in this category, making it the category with the highest number of responding participants and metaphors produced. “Wood” (f: 4) and “cactus” (f: 4), the most repeated metaphors in this category, were produced by female and male nurses in equal numbers. Moreover, female nurses produced the metaphors of “electricity,” “electric wire,” “electric pole,” “patient who has taken sedatives,” “baby,” “winter,” “stinging nettle,” “wall,” “gorilla,” “tiger,” and “fire.” Male nurses produced the additional metaphors of “bear” (f: 2), “fire,” “tough,” “truck driver,” “stone,” “street animal,” “trouble,” “uncooked meat,” “house that requires renovation,” “seawater,” “match,” and “patient prone to substance abuse and violence.”

A male patient is like a stinging nettle because he is very angry and offensive (FN, 4).

A male patient is like an electric pole because of his tense, angry, and pedantic attitude (FN, 14).

A male patient is like a gorilla because he has a rude appearance and manners (FN, 54).

A male patient is like wood because he is rude and tough. He doesn't understand courtesy (FN, 68).

A male patient is like a tiger because he becomes aggressive at the slightest problem (FN, 70).

A male patient is like a cactus because he becomes aggressive and unbearable when you touch him (FN, 77).

A male patient is like a match because he is easily inflammable but burns out quickly (MN, 97).

A male patient is like a patient prone to substance abuse and violence because he is angry and irritable (MN, 115).

A male patient is like a street animal because he lacks courtesy and is rude (MN, 130).

A male patient is like uncooked meat because he is tough (MN, 133).

A male patient is like a house that requires renovation because he always causes problems (MN, 135).

#### 4.2.5. Category 5. Irresponsible and Not Knowing His Limits

Three nurse clinicians produced 3 metaphors in this category. Female nurses produced the metaphors of “carefree” and “addicted to smoking,” while male nurses produced the metaphor of “ivy.”

A male patient is like a person addicted to smoking because he smokes everywhere without respecting the rules (FN, 6).

A male patient is like an ivy because he doesn't know his limits (MN, 91).

#### 4.2.6. Category 6. Difficult to Understand and Unable to Express Himself

Twenty-seven nurse clinicians produced 23 metaphors in this category, making this the category with the second-highest number of responding participants and metaphors produced. “Wood” (f: 4), the most repeated metaphor in this category, was produced by female nurses. Female nurses also produced the following metaphors: “book,” “cloud,” “bomb with its pin pulled,” “maze,” “wall,” “nail,” “stone,” “closed box,” “season,” “wavy sea,” “blank wall,” “refrigerator,” “door,” and “timid bird.” Male nurses also produced some additional metaphors: “book,” “treasure,” “wall that is difficult to overcome,” “red Ferrari car,” “video,” “night,” “box,” “chameleon,” and “bread.”

A male patient is like a season because it is unclear what he will do and when (FN, 1).

A male patient is like a blank wall because he is very hard and dull (FN, 2).

A male patient is like a wall because he looks at your face blankly when you talk to him. When you smile, he doesn't react. When you react, he reacts harshly (FN, 26).

A male patient is like a bomb with its pin pulled because it is unclear how they will react or what they will say (FN, 58).

A male patient is like wood because sometimes we explain, but he doesn't understand (FN, 78).

A male patient is like a book because he is better understood during reading (MN, 101).

A male patient is like a red Ferrari car because you look at it from the outside with great admiration, but when you sit inside, you realize its shortcomings (MN, 112).

A male patient is like a video because you cannot guess its content (MN, 127).

A male patient is like night because it is dark (MN, 132).

#### 4.2.7. Category 7. Delicate, in Need of Attention and Help, and Fragile

Nineteen nurse clinicians produced 14 metaphors in this category. “Child” (f: 4), the most repeated metaphor in this category, was produced by an equal number of female and male participants. Female nurses also produced the metaphors of “baby” (f: 2), “coy woman,” “hyena,” “cactus,” “mechanical pencil,” and “glass.” Male nurses produced the metaphors of “cat” (f: 2), “flower,” “brother,” “princess,” “hypochondriac,” “rubble,” and “brave lion.”

A male patient is like glass because he immediately collapses and is hurt at the slightest negativity (FN, 9).

A male patient is like a cactus because he needs more attention than a woman, although he looks tough and strong (FN, 28).

A male patient is like a coy woman because they become extremely coy and grumpy (FN, 42).

A male patient is like a flower because he is delicate (MN, 89).

A male patient is like a cat because he needs attention (MN, 106).

A male patient is like a princess because he is very delicate (MN, 137).

#### 4.2.8. Category 8. Not Thinking of Others

Five nurse clinicians produced four metaphors in this category. Female nurses produced the repeated metaphor “wood” (f: 2), along with “selfish” and “soulless.” Male nurses produced the metaphor of “the only patient in the world.”

A male patient is like a soulless person because they are selfish (FN, 48).

A male patient is like the only patient in the world because he doesn't think of others (MN, 103).

#### 4.2.9. Category 9. Strong, but Loses His Strength When Sick

Five nurse clinicians produced five metaphors in this category. There were no repeated metaphors. Female nurses produced the metaphors of “cat,” “person in bearskin,” and “fallen plane tree,” while male nurses produced the metaphors of “tree about to fall down” and “old weak tree that looks strong.”

A male patient is like a fallen plane tree because men are strong like plane trees. But they lose their strength when they get sick (FN, 50).

A male patient is like an old weak tree that looks strong because he always tends to appear strong but has many weaknesses due to male psychology (MN, 146).

#### 4.2.10. Category 10. The Patient has No Gender

Eight nurse clinicians produced three metaphors in this category. “Patient” (f: 6), the most used metaphor in this category, was mostly produced by male nurses (f: 4). Furthermore, in this category, the metaphor of “baby” was produced by a female nurse, and the metaphor of “my own child” was produced by a male nurse.

A male patient is like a baby because we care for and support him the same way we care for a child or baby. I have the same opinion for female and male patients (FN, 32)

A male patient is like a patient because I look at him like a patient, not like a man (FN, 65).

A male patient is like a patient because the patient has no gender (MN, 100).

A male patient is like my own child because you love him, get angry with him, but you have to fulfill your responsibilities toward him (MN, 139).

#### 4.2.11. Category 11. Fond of Comfort and Exaggerates His Illness

Three nurse clinicians, all of whom were female, produced three metaphors in this category: “cat,” “delicate,” and “magnifying glass.”

A male patient is like a cat because he constantly demands attention and feels like his world is ruined (FN, 29).

A male patient is like a magnifying glass because he makes small problems bigger (FN, 60).

#### 4.2.12. Category 12. Ungrateful

Two nurse clinicians, both of whom were female, produced two metaphors in this category: “hospital managers” and “cat.”

A male patient is like a cat because he is] ungrateful (FN, 12).

Male patients are like hospital managers because they behave ungratefully in case of even a small negligence (FN, 33).

#### 4.2.13. Category 13. Whose Hygiene/Care Is a Burden

Four nurse clinicians, all of whom were female, produced four metaphors in this category: “pantry,” “naughty boy,” “black,” and “suitcase.”

A male patient is like a pantry because his hygiene and care are challenging (FN, 41).

A male patient is like a suitcase because he is a burden (FN, 63).

#### 4.2.14. Category 14. Not Admitting His Illness

Three nurse clinicians, all of whom were male, produced three metaphors in this category: “culprit,” “ostrich,” and “naughty child.”

A male patient is like a culprit because he can deny the illness (MN, 83).

A male patient is like a naughty child because he doesn't admit his weakness, his illness, and doesn't listen to his relatives due to being raised in a patriarchal society. He expects absolute attention from the nurse or doctor (MN, 126).

#### 4.2.15. Category 15. Wanting to Recover as Soon as Possible

Four nurse clinicians produced three metaphors in this category. Both female and male nurses produced the repeated metaphor “impatient” (f: 2). In addition, male nurses produced the metaphors of “little child” and “high ego.”

A male patient is like a little child because he is very impatient and wants everything to happen at once (MN, 88).

A male patient is like an impatient person because he is stronger but expects immediate treatment (MN, 98).

## 5. Discussion

The present study aimed to analyze the presence of gender stereotypes in the metaphors that nurse clinicians use to describe patients. In line with the research questions, the findings are discussed as follows.

### 5.1. Discussion on Gender Stereotypes and Nurse Clinicians' Metaphors for Their Patients

Other studies using metaphor analysis to examine gender stereotypes in nurse clinicians' perceptions of their patients could not be found. Existing research on the gender perceptions of nurse clinicians largely consists of quantitative studies, most of which suggest that nurses have egalitarian gender perceptions [2, 4, 7, 10, 11, 13, 14, 19]. However, contrary to these studies, other studies have determined that nurses do have gender stereotypes [4, 11]. One of these studies was conducted with 200 nurses who worked at a training and research hospital; 44% of the nurses stated that the best aspect of being male was being free, and 64.5% stated that the best aspect of being female was being a mother. Furthermore, approximately half of the nurses in the study expressed violence as a gender-specific behavior that men should not exhibit, while 26.5% indicated infidelity as a behavior that women should not exhibit. Furthermore, 50.5% of the nurses stated that men should be the breadwinners of the family, while 79.5% indicated that both mothers and fathers should care for and raise their children [11]. A society with a traditional patriarchal culture defines men as strong, violent, and free, while describing women as passive, loyal, and honorable [20]. Findings from various studies indicate that the culture the nurses were raised in may have an effect on their adoption of traditional roles [11]. As seen from the findings of this study, nurses mainly used metaphors in the “plant” and “qualitative” categories when describing female patients. On the other hand, metaphors in the “object” and “qualitative” categories were primarily used when describing male patients. Nurses most frequently used the “flower” (23) metaphor for female patients and the “wood” (10) metaphor for male patients. In the “plant” category, nurses produced the metaphors of “wilted flower,” “withered rose,” “red flower,” and “daisy” for female patients, while the metaphor of “cactus” (f: 7) was most used for male patients. In the “animal” category, nurses produced the metaphors of “rabbit” (f: 3), “cat,” “swan,” “gazelle,” “butterfly,” and “wounded bird” for female patients, while the “hyena,” “lion,” “gorilla,” “tiger,” and “bear” (f: 2) metaphors were most used for male patients. The nurses mostly described female patients as “delicate, in need of attention and help, and fragile” and “tough, dissatisfied, and angry.” On the other hand, the nurses mostly described male patients as “strong, authoritative, head of the family, and resilient,” “rude, dissatisfied, and angry,” and “difficult to understand and unable to express himself.”

Finally, a total of eight nurses produced metaphors for both female and male patients, describing them as “the patient has no gender.”

Upon examining the nurse clinicians' metaphors and metaphor explanations, it can be said that most of the nurses held gender stereotypes. These stereotypes were reflected in their perceptions of their patients. Existing literature has reported that gender stereotypes affect gender-related metaphors, causing stereotypes about women and men to continue to remain in existence [21]. The results of this study are in line with this literature.

### 5.2. Discussion on Gender Stereotypes and Nurse Clinicians' Metaphors for Patients by Gender

Many studies on nurses' gender perceptions and associated factors have found that female nurses have more egalitarian attitudes toward gender roles than male nurses [4, 7, 22]. A review of studies on the subject revealed that researchers have mostly examined the gender perceptions of nursing students; most of these studies find that women exhibit a more egalitarian attitude toward gender roles than men [8, 23–26]. On the contrary, some studies have determined that men have more egalitarian gender perceptions than women [27–29]. Among these is the qualitative study conducted by Ünal Toprak and Turan (2021) on the gender roles of nursing students; female students were asked about their opinions on the “role of being a woman,” while male students were asked about their opinions on the “role of being a man.” The researchers asked the nursing students to criticize the situation in social life. Female students emphasized negative situations such as women “being exposed to violence, being oppressed, being interfered with in their clothing, and being forced to serve their family members constantly.” Male students, on the other hand, stressed that men assume significant responsibilities in exchange for their social comfort [29].

In the present study, both female nurses (n: 33) and male nurses (n: 29) generally described female patients as “delicate, in need of attention and help, and fragile.” However, mostly female nurses (n: 16) used “flower” (f:2 3), the most frequently repeated metaphor, for female patients. Mostly female nurses (n: 12) described female patients as “harsh, dissatisfied, and angry.” For male patients, both female nurses and male nurses mostly produced metaphors describing male patients as “strong, authoritative, head of the family, and resilient,” “rude, dissatisfied, and angry,” and “difficult to understand and unable to express himself.” Mostly female nurses (f: 8) used the frequently repeated metaphor “wood” (f: 10) for male patients. However, only male nurses described female patients as “private,” and only female nurses described female patients as “precious, fun, and beautiful.” A female nurse likened a male patient's delicacy to a “delicate woman.” A male nurse described a male patient's strength with the metaphor of “man.” Both female and male nurses associated coyness with women and strength with men. Finally, a total of eight nurses, including five male nurses and three female nurses, produced three metaphors for both female and male patients, using the description “the patient has no gender.” The most used metaphor in this category, “patient” (f: 6), was mostly used by male nurses (f: 4). It is seen that previous studies have obtained differing findings, which may be a result of the cultural structures of the societies where individuals are raised or the upbringing styles of the families [8, 27].

## 6. Limitations of the Study

The findings of the current study are based on the metaphors produced by 149 nurse clinicians. Accordingly, the study provides partial information on how gender stereotypes affect nursing practice.

## 7. Conclusion

The nurse clinicians in this study generally described female patients as in need of more care, love, and compassion. On the other hand, the nurse clinicians generally described male patients as rude, tough, and strong. Five male nurses and three female nurses stated that a patient has no gender. These nurses viewed female and male patients as patients, regardless of their gender.

In the literature, metaphor is defined as the way people see the world and derive meaning out of it [30]. In addition, it is mentioned that metaphor reveals thoughts and perceptions that individuals are not aware of [31]. However, it is stated that conceptual metaphors are based on biological, cultural, interactional, and even neurological experiences that we perceive in different ways and that these experiences motivate metaphors [32, 33]. Meaningfully, it is important not to overlook the factors underlying these metaphors produced by nurse clinicians. Moreover, extensive studies are needed to take into account additional factors such as cultural background, experience, or specific patient interactions that may have a major impact on how nurse clinicians perceive gender.

## 8. Implications for Nursing Management

This study evaluates gender stereotypes in nurse clinicians' perceptions of their patients, and it highlights some important points in terms of nursing and patient care. In the study, it can be seen that the nurse clinicians held gender stereotypes with their patients.

## Figures and Tables

**Table 1:** Descriptive characteristics of nurse clinicians (*N*: 149).

Variables		Frequency of the variable (%)	Min–max	Mean ± SD
Gender	Female	82 (55)		
	Male	67 (45)		

Age range	22–29 years	80 (53.7)		
	30–39 years	49 (32.9)		
	40 and above	20 (13.4)		

Age (mean ± SD)			22–50	31.14 ± 6.826

Marital status	Married	88 (59.1)		
	Single	58 (38.9)		
	Divorced	3 (2)		

Number of children	No children	78 (52.3)		
	1	24 (16.1)		
	2	37 (24.8)		
	3 and more	10 (6.7)		

Educational background	Medical vocational high school	31 (20.8)		
	University	105 (70.5)		
	Postgraduate	13 (8.7)		

Ward of employment	Intensive care	43 (28.9)		
	Adult emergency	1 (0.7)		
	Surgical clinics	46 (30.9)		
	Internal medicine clinics	45 (30.2)		
	Outpatient clinic	14 (9.4)		

Working years range	1–5 years	47 (31.5)		
	6–10 years	53 (35.6)		
	11–15 years	22 (14.8)		
	16 years and more	27 (18.1)		

Working years (mean ± SD)			1–32	9.54 ± 6.466

**Table 2:** The metaphors and their categories used by nurse clinicians for a “female patient”/categories of metaphor explanations used by nurse clinicians for a “female patient.”

**Number**	**Category**	**Metaphors used by female nurses**	**Metaphors used by male nurses**	**F**

1	Human	Baby (3); child; girl (2); newborn	Baby (2); child (2); newborn	4
2	Animal	Rabbit (2); lion; cat; ostrich; swan	Rabbit; ant; butterfly; gazelle; wounded bird; bird with a broken wing; bird; wounded lion	12
3	Plant	Flower (16); cactus (2); tree; withered rose; lettuce; four o'clock flower; cotton; cabbage; branch; fruit tree; thorn	Flower (7); cactus; tree; withered rose; wilted flower (2); daisy; fallen leaf; sapling; red flower; ∗inverted tulip	17
4	Nature	Cloud; stream bed; winter; earth; sky; mountain (2)	Water	8
5	Qualitative	Delicate flower; dissatisfied; poor; oppressed (2); sacred being; suicide; suffering (2); white; psychiatric patients	Delicate flower; burden; sensitive (2); torture; irregular; low pain threshold; fair; trouble; fragile flower; patient with depression; hypochondriac	19
6	Symbol	Glass ceiling; game; fire; patience stone	Poison; time	6
7	Object	Dynamite ready to explode; glass; ballpoint pen; tape recorder; matryoshka; pressure cooker; vase	Glass (3); car; bell jar; candle; porcelain; toothpick; mirror	14
8	Role	Mother/my mother (5); patient (2); aunt; beautiful mother; mother-in-law; elder sister; naughty child	Mother/my mother (3); patient (4); aunt; my own child; new bride; coy child; child whose toy was taken away	11
9	Food/beverage	Tulumba dessert	Ashura	2

**Number**	**Categories of metaphor explanations**	**Metaphors Used by Female Nurses**	**Metaphors Used by Male Nurses**	**F**

1	Family member	My mother/mother (3); aunt; elder sister	My mother/mother (2); aunt	4
2	Delicate, in need of attention and help, and fragile	Baby (2); child; flower (14); newborn; fruit tree, branch; patience stone; oppressed; glass; girl (2); four o'clock flower; rabbit (2); withered rose; vase; delicate flower; cloud; cactus	Baby (2); child; flower (6); delicate flower; newborn; fragile flower; wilted flower; child whose toy was taken away; sensitive; daisy; sapling; glass (3); butterfly; low pain threshold; tree; car; new bride; gazelle; wounded bird; patient with depression; toothpick	33
3	Thinking about her responsibilities at home but neglecting herself	Poor; oppressed; suffering (2); mother	Rabbit; wilted flower; wounded lion; candle; porcelain	9
4	Dressed up in layers	Lettuce; cabbage	—	2
5	Keeping alive and directing	Earth; tree; stream bed	Bird; ant	5
6	Precious, fun, and beautiful	Sacred being; tulumba dessert; game; sky; swan; beautiful mother	—	6
7	Strong and resilient	Mountain (2); lion; ballpoint pen; mother	Withered rose	5
8	Easy-going and leaving things to experts	Cotton; ostrich	Fair	3
9	Clean and active in her care	Flower (2); white	Flower	2
10	Harsh, dissatisfied, and angry	Cactus; winter; mother-in-law; dynamite ready to explode; dissatisfied; naughty child; cat; psychiatric patient; tape recorder; pressure cooker; fire; thorn	Cactus; water; torture; poison; coy child; hypochondriac	17
11	The patient has no gender	Patient (2); baby	Patient (4), my own child	3
12	Private	—	Sensitive; red flower; mother; inverted tulip	4
13	Weak and unable to recover when sick	Suicide	Fallen leaf, bird with a broken wing	3
14	Difficult to understand and unable to express herself	Glass ceiling; matryoshka	Bell jar; time; trouble; ashura	6
15	Other	—	Mirror (behaves in line with your approach toward her.); burden (overweight); irregular (does not pay attention to hygiene.)	3

*Note*: The flowers of this plant, also known as the weeping bride in the relevant geography, have different colors and point toward the ground, unlike tulips [18].

^∗^Inverted tulip: it is an endemic flower species belonging to Anatolian geography.

**Table 3:** The metaphors and their categories used by nurse clinicians for a “male patient”/categories of metaphor explanations used by nurse clinicians for a “male patient.”

**Number**	**Category**	**Metaphors used by female nurses**	**Metaphors used by male nurses**	**F**

1	Human	Child (2); baby (4)	Child (3)	2
2	Animal	Chameleon; cat (3); hyena; lion (2); gorilla; tiger	Chameleon; cat (2); bear (2); ant; ostrich	9
3	Plant	Cactus (4); stinging nettle; fallen plane tree	Cactus (3); flower; ivy; tree about to fall down	6
4	Nature	Stone (2); mountain; winter; season; wavy sea; cloud	Stone; mountain; seawater	7
5	Qualitative	Impatient; positive motivation; authority; carefree; selfish; bully; soulless; delicate; person in bearskin; black; timid bird; patient who has taken sedatives	Impatient; an old weak tree that looks strong; tough; gentleman; problem; night; resilient; the only patient in the world; dead; high ego; man; fellow man; brave lion; ignorant wise man; street animal; patient prone to substance abuse and violence; hypochondriac	28
6	Symbol	Maze; electricity; fire	Google; fire; treasure; video	7
7	Object	Wood (8); book; iron; refrigerator; suitcase; electric wire; nail; blank wall/wall (3); mechanical pencil; door; electric pole; pine; bomb with its pin pulled; magnifying glass; closed box	Wood (2); book; iron; rubble; wall that is difficult to overcome; match; column of the building; red Ferrari car; box	21
8	Role	Father/my father (3); patient (2); hospital managers; elder brother; my grandfather (2), uncle; strong father; addicted to smoking; naughty boy; coy woman	Father/my father (2); patient (4); culprit; truck driver; brother; princess; my own child; naughty child	16
9	Food/beverage	—	Sugar; uncooked meat; bread; honey	4
10	Place	Pantry	House that requires renovation	2

**Number**	**Categories of metaphor explanations**	**Metaphors used by female nurses**	**Metaphors used by male nurses**	**F**

1	Family member	Father/my father (3); grandfather/my grandfather (2); uncle	Father/my father	3
2	Easy-going and leaving things to experts	Positive motivation; chameleon	Gentleman; sugar; dead; fellow man; honey	7
3	Strong, authoritative, head of the family, and resilient	Iron; cactus; mountain; authority; bully; elder brother; strong father; stone; lion (2)	Iron; cactus; mountain; resilient; ant; man; father; google; ignorant wise man; column of the building	16
4	Rude, dissatisfied, and angry	Wood (2); cactus (2); electricity; electric wire; electric pole; patient who has taken sedatives; baby; winter; stinging nettle; wall; gorilla; tiger; fire	Wood (2); cactus (2); bear (2); fire; tough; truck driver; stone; street animal; problem; uncooked meat; house that requires renovation; seawater; match; patient prone to substance abuse and violence	25
5	Irresponsible and not knowing his limits	Carefree, addicted to smoking	Ivy	3
6	Difficult to understand and unable to express himself	Book; wood (4); cloud; bomb with its pin pulled; maze; wall; nail; stone; closed box; season; wavy sea; blank wall; refrigerator; door; timid bird	Book; treasure; wall that is difficult to overcome; red Ferrari car; video; night; box; chameleon; bread	23
7	Delicate, in need of attention and help, and fragile	Child (2); baby (2); coy woman; hyena; cactus; mechanical pencil; glass	Child (2); cat (2); flower; brother; princess; hypochondriac; rubble; brave lion	14
8	Not thinking of others	Wood (2); selfish; soulless	The only patient in the world	4
9	Strong, but losing his strength when sick	Cat, person in bearskin; fallen plane tree	Tree about to fall down; an old weak tree that looks strong	5
10	The patient has no gender	Patient (2); baby	Patient (4), my own child	3
11	Fond of comfort and exaggerates his illness	Cat; delicate; magnifying glass	—	3
12	Ungrateful	Hospital managers; cat	—	2
13	Whose hygiene/care is a burden	Pantry; naughty boy; black; suitcase	—	4
14	Not admitting his illness	—	Culprit; ostrich; naughty child	3
15	Wanting to recover as soon as possible	Impatient	Little child; impatient; high ego	3

## Data Availability

The authors elect to not share data.
